# Differential Resistance of *Borrelia burgdorferi* Clones to Human Serum-Mediated Killing Does Not Correspond to Their Predicted Invasiveness

**DOI:** 10.3390/pathogens12101238

**Published:** 2023-10-13

**Authors:** Patrick Pearson, Connor Rich, Eric L. Siegel, Dustin Brisson, Stephen M. Rich

**Affiliations:** 1 Department of Microbiology, University of Massachusetts Amherst, Amherst, MA 01003, USA; pbpearson@umass.edu (P.P.); crich@umass.edu (C.R.); esiegel@umass.edu (E.L.S.); 2 Department of Biology, University of Pennsylvania, Philadelphia, PA 19104, USA; dbrisson@sas.upenn.edu

**Keywords:** *Borrelia burgdorferi*, human serum, host associations, complement

## Abstract

Reservoir host associations have been observed among and within *Borrelia* genospecies, and host complement-mediated killing is a major determinant in these interactions. In North America, only a subset of *Borrelia burgdorferi* lineages cause the majority of disseminated infections in humans. We hypothesize that differential resistance to human complement-mediated killing may be a major phenotypic determinant of whether a lineage can establish systemic infection. As a corollary, we hypothesize that borreliacidal action may differ among human subjects. To test these hypotheses, we isolated primary *B. burgdorferi* clones from field-collected ticks and determined whether the killing effects of human serum differed among those clones in vitro and/or whether these effects were consistent among human sera. Clones associated with human invasiveness did not show higher survival in human serum compared to noninvasive clones. These results indicate that differential complement-mediated killing of *B. burgdorferi* lineages is not a determinant of invasiveness in humans. Only one significant difference in the survivorship of individual clones incubated in different human sera was detected, suggesting that complement-mediated killing of *B. burgdorferi* is usually similar among humans. Mechanisms other than differential human complement-mediated killing of *B. burgdorferi* lineages likely explain why only certain lineages cause the majority of disseminated human infections.

## 1. Introduction

Host associations exist between and within genospecies of the *Borrelia burgdorferi* sensu lato complex [1,2,3,4,5]. Differential resistance to the killing effects of host serum is a major determinant of host associations among *Borrelia* genospecies [1,2,4]. The European genospecies *B. afzelii* and *B. garinii* are considered rodent-associated and avian-associated, respectively [2,6,7]. Particular *Borrelia* genospecies can survive in the presence of serum from the taxonomic class of hosts they typically infect but not others [1,2,4,8]. The borreliacidal activity of host serum is due to the effects of the complement system, a component of host innate immunity [2,9]. The complement system is a network of proteins which clears invading pathogens by facilitating phagocytosis and directly killing cells via the formation of a membrane attack complex [10,11]. We and others suspect that host associations among variants of an individual *Borrelia* species may also be due to differential serum (complement)-mediated killing [12].

*Borrelia burgdorferi* sensu stricto (hereafter *B. burgdorferi*) is a tick-borne human pathogen with observed host associations among its diverse lineages [5,13]. There are at least 25 different *B. burgdorferi* lineages in the United States which can be distinguished by the nucleotide sequences encoding the outer surface protein C (OspC) [5,14,15]. Experiments using laboratory animals have demonstrated that some *B. burgdorferi* lineages have a higher fitness in experimentally infected animals [16,17,18]. Field experiments have shown that reservoir hosts are commonly infected with unique subsets of the *B. burgdorferi* lineages [5,19,20,21]. Humans are no exception to this pattern, as the majority of human-disseminated infections are caused by a small subset of *B. burgdorferi* lineages [22,23,24,25]. The *B. burgdorferi* lineages characterized by *ospC* genotypes A, B, I, K, and N are considered human-invasive, and the other lineages rarely disseminate in humans [19,22,23,24,25,26].

The observed host preferences by different *B. burgdorferi* lineages may be mediated by differential resistance to host complement. This idea is supported by recent experiments demonstrating that differential resistance to killing by host serum is a factor contributing to the fitness of different strains within *B. burgdorferi* in laboratory animals [12,27]. *B. burgdorferi* has a multifunctional and redundant set of anti-complement outer surface proteins [2,3,9]. The anti-complement proteins can be variable among *B. burgdorferi* strains and confer strain-specific survival differences in host serum [3,12,27,28]. It is expected that *B. burgdorferi* lineages that efficiently infect a particular host will survive better in serum collected from that host compared to other *B. burgdorferi* lineages. 

In this study, we focused on the observed associations between humans and *B. burgdorferi* lineages. We determined whether human-invasive clones have higher survival probabilities compared to human noninvasive clones and if these effects are consistent among human sera. *B. burgdorferi* is considered to be resistant to the borreliacidal activity of human serum [1,2,8,9,29]. However, laboratory strains within *B. burgdorferi* have typically been used in similar experiments, and survival in human serum has been found to vary widely among strains [8,30,31,32,33,34,35]. We used a set of low-passage, tick-derived *B. burgdorferi* clones characterized by their *ospC* sequence. The *B. burgdorferi* cultures used in these experiments better represent the spirochetes potentially transmitted to humans by feeding ticks.

## 2. Materials and Methods

### 2.1. B. burgdorferi ospC Clone Isolation and Characterization

*B. burgdorferi* clones were isolated directly from field-collected *Ixodes scapularis* ticks by limiting dilution and were frozen at −80 °C [36,37] (File S1). The clones were genotyped based on their outer surface protein C (*ospC*) sequence using Luminex technology (Austin, TX, USA), as previously described [38], and by Sanger sequencing. No mixed *ospC* sequences were detected by either method. The clones used in this study represent two invasive *B. burgdorferi* lineages (*ospC* genotypes A and I) and three noninvasive lineages (*ospC* genotypes G, H, and M). Hereafter, they are referred to as clones-A, -I, -G, -H, or -M. The actual invasiveness to humans of the isolated clones cannot be verified, but their predicted invasiveness is based on previous research [19,22,23,24,25]. A growth curve was performed for each clone prior to the serum sensitivity assay (File S1, Figure S1). 

### 2.2. Human Serum Collection and Validation

Human blood was collected from two males and one female, hereafter referred to as PP, CR, and LJ, respectively. This study did not meet the definition of human subject research, and IRB approval was not required. The blood was collected in serum separator tubes, allowed to clot at room temperature for up to 45 min and then centrifuged at 1500 RCF for 10 min to separate serum. Sera from multiple tubes from each donor were pooled, filtered through a 0.22 um filter, and stored in 0.5 mL aliquots at −80 °C. Aliquots were tested for a functional alternative complement pathway using the Total Complement Functional Screen ELISA kit (Eagle Biosciences Inc., Amherst, NH, USA) to ensure complement activity was retained in the collected serum. One aliquot of human serum was heat-inactivated at 56 °C for 30 min before testing. Complement activity was detected in serum collected from each donor, while complement activity was completely abolished in the heat-inactivated serum aliquot. All blood donors were negative for anti-*B. burgdorferi* antibodies, as determined by an immunoassay performed at a commercial laboratory (Labcorp., Enfield, CT, USA).

### 2.3. Serum Sensitivity Assay

The sensitivity of the *B. burgdorferi* clones to human serum was measured as previously described [39]. The clones were grown in BSK-H medium with 6% rabbit serum (BSK-H complete, Sigma) at 34 °C to mid-logarithmic phase and diluted to 3 × 10^6^ cells/mL in BSK-H media without rabbit serum (BSK-H incomplete, Sigma, St. Louis, MO, USA). Then, 400 μL of human serum was added to 400 μL of culture (yielding 50% human serum concentration), and 200 μL of this mixture was aliquoted into triplicate wells on a 96-well plate and incubated at 34 °C for 4 h. BSK-H incomplete was substituted for 50% human serum as a positive control. Viable cells were counted by darkfield microscopy at 0 and 4 h. Viable cells were defined as those that were visibly motile and retained a characteristic spirochete shape [39,40]. The percent survival was calculated as the number of viable cells after 4 h divided by the number of viable cells at the 0-h time point multiplied by 100. To determine whether killing was complement-mediated, we also tested the sensitivity of clone-I in media supplemented with heat-inactivated (56 °C for 30 min) human serum from LJ. The survival of clone-I in normal and heat-inactivated LJ serum was tested in a separate experiment to the one testing the survival of clone-I in PP and CR sera, and the positive control was repeated (*n* = 6 total replicates for positive control). 

The same assay was performed using the clones mixed with 50% white-tailed deer serum (*Odocoileus virginianus*) as a control to observe and validate the serum-mediated killing of spirochetes [39]. The killing of spirochetes by white-tailed deer serum was confirmed by passaging an aliquot of each positive control and test sample at the 4-h time point to fresh BSK-H complete media and incubating at 34 °C. The subcultures were incubated for 6–8 days and checked by darkfield microscopy for the presence of *B. burgdorferi* growth [39]. 

Morphologically heterogenous and motile cells were also observed during the 4-h serum sensitivity assay (File S2, Figures S2–S5). The inclusion of these cells in a separate analysis did not change the conclusions of this study (File S2, Figure S6, Table S1).

### 2.4. Statistical Analysis

The percent survival values for the clones incubated in the positive control and human serum were log-transformed and checked for the assumptions of normality and homoscedasticity. Significant differences in the survival of the clones were detected using a two-way ANOVA, and multiple comparisons were corrected using a Tukey’s test in Prism version 9.0.0 (Graphpad Software LLC., San Diego, CA, USA). *p*-values of less than 0.05 were considered significant. 

## 3. Results

**Figure 1:**
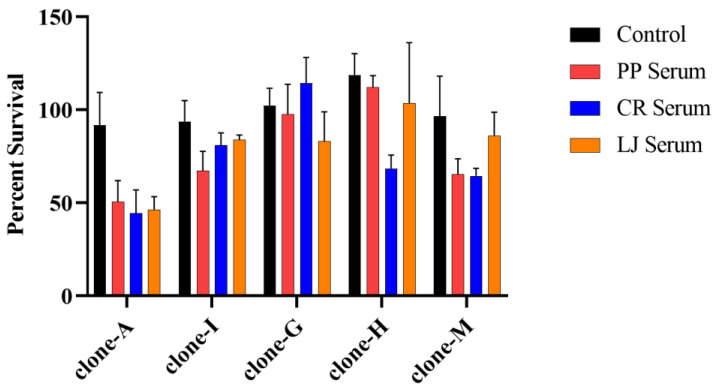
Survivorship of two human-invasive clones (clones-A and -I) and three noninvasive clones (clones-G, -H, and -M) in human serum. Bars represent mean values from three or six replicates, and error bars represent standard deviation. Significant differences determined from log-transformed values using a two-way ANOVA and Tukey’s multiple comparisons test are shown in Table 1.

The five clones survived in human serum (Figure 1) but were effectively killed by white-tailed deer serum, as previously reported [39]. No viable spirochetes were observed for any clone after a 4-h incubation in white-tailed deer serum, and no growth in subculture was detected on days 6–8 [39]. The survival of some clones incubated in human serum was significantly lower compared to survival in the positive control (Figure 1, Table 1). The serum in this study retained complement activity, and clone-I had a higher survival when incubated in heat-inactivated versus normal sera collected from LJ (Figure S7). 

Differential survival among the *B. burgdorferi* clones was observed, but it did not conform to expectations based on the clones’ human-invasive or -noninvasive designation. It was expected that invasive clones-A and -I would have a significantly higher survivorship compared to the noninvasive clones -G, -H, and -M. However, clone-A had the lowest percent survival values, while clone-I had a survivorship similar to clones-G, -H, and-M when incubated in human serum (Figure 1). Several pairwise comparisons indicated a significantly lower survivorship of an invasive clone compared to a noninvasive clone (Table 1). The overwhelming majority of these pairwise differences (*n* = 18) included clone-A. None of these pairwise comparisons yielded a significantly higher survivorship of an invasive clone compared to a noninvasive clone. 

The survivorship of individual clones incubated in human sera (PP, CR, LJ) was generally homogenous (Figure 1). The percent survival values of clones-A and -I incubated in different human sera were similar and ranged from 44.4–50.7% and 67.2–83.8%, respectively. Clone-G had a higher level of survival in the serum from one individual (CR) compared to sera from other donors. The percent survival of clone-M incubated in LJ serum was 86.2%, whereas only approximately 65% of clone-M spirochetes survived in sera collected from PP and CR. These differences were not significant, however. Only one significant difference was noted between clone-H incubated in PP and CR sera (Figure 1, Table 1). 

## 4. Discussion

We hypothesized that differential resistance to human complement may be a determinant of differential human infectivity among *B. burgdorferi* lineages. Reservoir host associations exist among *Borrelia* genospecies, and these associations are maintained by differential host-serum-mediated killing [1,2,4]. The model of serum-mediated host associations has been extended from among *Borrelia* species to strains within a single species, *B. burgdorferi* [12,27,41]. Preference for human hosts has also been reported to differ among certain *B. burgdorferi* lineages [22,23,24,25]. We tested whether *B. burgdorferi* lineages associated with human-invasive and -noninvasive infections had different survival rates in media supplemented with human serum.

These data do not support the hypothesis that the invasive *B. burgdorferi* lineages are more resistant to human-complement-mediated killing compared to the lineages that rarely disseminate in humans. Previous research has demonstrated that *B. burgdorferi* is resistant to human-serum-mediated killing but that variation can occur among strains within *B. burgdorferi* [1,2,29,31,34]. Our results agree, since every *B. burgdorferi* clone survived, and significant differences in survivorship were detected among the clones. The observed borreliacidal activity was likely complement-mediated, but we cannot exclude the possibility of other heat-sensitive factors. The survival differences, however, did not correspond to whether the clones are considered invasive or noninvasive in humans as defined by their *ospC* genotype. We used a single clone to represent a *B. burgdorferi* lineage, but spirochetes within a *B. burgdorferi* lineage can have different serum-survival phenotypes and invasiveness levels in hosts [12,21,22,27]. Determining the resistance to human serum of several clones with the same *ospC* genotype may help resolve this potential concern. 

We speculated that the survival of a clone in human serum may be impacted by the specific individual from whom the serum was collected. Previous studies with similar assays have typically used pooled human serum from many individuals, serum collected from one donor, or do not clearly match serum-survival results with specific individuals if there are multiple donors [8,30,31,32,33,34,42]. These results show that the survival of a specific clone incubated in different human sera was similar, and only one significant difference was detected. An expanded study using sera collected from many individuals is needed to fully address this question. 

It is unclear why our data do not show a connection between the serum resistance of *B. burgdorferi* lineages and their predicted invasiveness in humans. Recent studies have reported *B. burgdorferi* strain-specific survival in different host sera [12,27,41]. The serum survival phenotype of the *B. burgdorferi* strains is a major factor affecting fitness in laboratory animals [12,27]. However, the serum resistance of *B. burgdorferi* strains does not always match their infectivity in hosts and is most likely due to complement-independent mechanisms [17,27]. For example, *B. burgdorferi* strain cN40 had a high survivorship in white-footed mouse (*Peromyscus leucopus)* serum but a low fitness in mice [27]. Wang et al. [17] also found no significant correlation between the serum resistance of individual *B. burgdorferi* strains and their capacity to cause disseminated infections in C3H/HeJ (*Mus musculus*) mice, although *M. musculus* complement is unstable in vitro, which may have affected these results [12,43]. 

Other factors besides human-complement-mediated killing are likely facilitating the differential propensity of certain *B. burgdorferi* lineages to infect humans. OspC may play a direct role, since it is polymorphic and interacts with several host molecules [15,44,45,46,47,48]. The differential binding affinities of the polymorphic OspC variants to their known human ligands may enable the human-invasive lineages to develop a systemic infection more frequently in humans. OspC is also an antiphagocytic factor, and the differential evasion of immune cells by spirochetes expressing different OspC variants is another potential explanation [49]. It is also possible that while the *ospC* gene can be used for defining *B. burgdorferi* lineages, other linked genetic elements likely have a greater impact on the invasiveness of lineages in humans [50]. Another possibility is that *B. burgdorferi* lineages do exhibit differential resistance to human-complement-mediated killing which corresponds to invasiveness, but these differences were not detected in our in vitro assay. 

We have demonstrated that *B. burgdorferi* clones representing canonical human-invasive lineages are not more resistant to human-complement-mediated killing. These results also show that human-complement-mediated borreliacidal activity does not usually differ among individuals. Additional research is necessary to understand the determinants of invasiveness in humans by different *B. burgdorferi* lineages. A more detailed understanding of the factors shaping associations between humans and *B. burgdorferi* lineages may lead to better Lyme disease management strategies. 

## Figures and Tables

**Table 1:** Results of two-way ANOVA with Tukey’s multiple comparisons test to determine survival differences of clones incubated in human serum and the control.

		Clone-A				Clone-I				Clone-G				Clone-H				Clone-M			
		Ctrl	PP	CR	LJ	Ctrl	PP	CR	LJ	Ctrl	PP	CR	LJ	Ctrl	PP	CR	LJ	Ctrl	PP	CR	LJ
clone-A	Ctrl		**	***	***	ns	ns	ns	ns	ns	ns	ns	ns	ns	ns	ns	ns	ns	ns	ns	ns
	PP			ns	ns	***	ns	ns	*	***	**	****	*	****	****	ns	***	**	ns	ns	*
	CR				ns	****	ns	**	**	****	****	****	**	****	****	ns	****	****	ns	ns	***
	LJ					****	ns	*	**	****	***	****	**	****	****	ns	****	***	ns	ns	**
clone-I	Ctrl						ns	ns	ns	ns	ns	ns	ns	ns	ns	ns	ns	ns	ns	ns	ns
	PP							ns	ns	ns	ns	*	ns	*	*	ns	ns	ns	ns	ns	ns
	CR								ns	ns	ns	ns	ns	ns	ns	ns	ns	ns	ns	ns	ns
	LJ									ns	ns	ns	ns	ns	ns	ns	ns	ns	ns	ns	ns
clone-G	Ctrl										ns	ns	ns	ns	ns	ns	ns	ns	ns	ns	ns
	PP											ns	ns	ns	ns	ns	ns	ns	ns	ns	ns
	CR												ns	ns	ns	*	ns	ns	*	*	ns
	LJ													ns	ns	ns	ns	ns	ns	ns	ns
clone-H	Ctrl														ns	*	ns	ns	**	**	ns
	PP															*	ns	ns	*	*	ns
	CR																ns	ns	ns	ns	ns
	LJ																	ns	ns	ns	ns
clone-M	Ctrl																		ns	ns	ns
	PP																			ns	ns
	CR																				ns
	LJ																				

The percent survival values for the clones incubated in the positive control and human serum were log-transformed, and significant differences were detected using a two-way ANOVA and Tukey’s multiple comparisons test. Significant differences (*n* = 50) out of 190 comparisons are denoted by asterisks. *p*-values ≤ 0.05, 0.01, 0.001, 0.0001 are denoted by *, **, ***, ****, respectively. ns—not significant; Ctrl—positive control.

## Data Availability

Data are available upon request.

## Supplementary Materials

The following supporting information can be downloaded at: https://www.mdpi.com/article/10.3390/pathogens12101238/s1. File S1: Supplementary Materials and methods. File S2: Observation and analysis of atypically shaped, motile cells of *B. burgdorferi* encountered during the 4-h serum sensitivity assay. Figure S1: Growth curves of *B. burgdorferi ospC* clones. Individual data points represent mean concentrations (*n* = 3 replicates), and error bars represent standard deviation. Figure S2: Representative image of a *B. burgdorferi* spirochete with normal cell morphology. Figure S3: First representative image of a *B. burgdorferi* cell with atypical morphology. This example is characterized by a ring-shaped cell. Figure S4: Second representative image of a *B. burgdorferi* cell with atypical cell morphology. This example is characterized by a cell with a coiled end. Figure S5: Third representative image of a *B. burgdorferi* cell with atypical cell morphology. This example is characterized by a cell with multiple coils or folds at one end. Figure S6: Survivorship of two human-invasive clones (clones-A and -I) and three noninvasive clones (clones-G, -H, and -M) in human serum when all motile cells are included. The percent survival values were calculated using all motile *B. burgdorferi* cells, regardless of whether they exhibited typical or atypical cell morphology. Bars represent mean values from three or six replicates, and error bars represent standard deviation. Significant differences determined using a two-way ANOVA and Tukey’s multiple comparisons test are included in Table S1. Figure S7: Survival of clone-I incubated in the control and in normal and heat-inactivated human serum. Bars represent mean percent survival (*n* = 3 replicates), and error bars represent standard deviation. Human serum collected from LJ. HI—heat-inactivated. Table S1: Results of two-way ANOVA with Tukey’s multiple comparisons test to determine survival differences of clones incubated in human serum and the control, using all motile cells. All motile cells regardless of morphology were considered viable in this analysis. The percent survival values for the clones incubated in the positive control and human serum were log-transformed, and significant differences were detected using a two-way ANOVA and Tukey’s multiple comparisons test. Significant differences (*n* = 9) out of 190 comparisons are denoted by asterisks. *p*-values ≤ 0.05, 0.01, 0.001, 0.0001 are denoted by *, **, ***, ****, respectively. NS—not significant; Ctrl—positive control.
