# Supplementary material for: Differential Resistance of Borrelia burgdorferi Clones to Human Serum-Mediated Killing Does Not Correspond to Their Predicted Invasiveness
Source: Pathogens. 2023 Oct 13;12(10):1238. doi: 10.3390/pathogens12101238 (PMC10609869; doi:10.3390/pathogens12101238)
Supplement: Supplementary file 1 [file pathogens-12-01238-s001.zip › File_S1.pdf]

SUPPLEMENTARY MATERIALS AND METHODS

Tick collection and *B. burgdorferi* primary isolates

Adult *Ixodes scapularis* ticks were collected by flagging vegetation [36] from sites in Massachusetts and Vermont. Collected ticks were kept live at 4-8°C in vials with high humidity. To obtain *B. burgdorferi* primary isolates, ticks were surface sterilized by three sequential, five-minute washes in 1) betadine, 2) hydrogen peroxide, and 3) 70% ethanol. Individual ticks were triturated in glass tissue homogenizers with BSK-H medium with 6% rabbit serum (BSK-H complete, Sigma) supplemented with 50 ug/ml rifampicin, 20 ug/ml phosphomycin, and 2.5 ug/ml amphotericin B [37]. Approximately 1.3 ml of the tick homogenate was then transferred to sterile 1.5 ml screw cap tubes and incubated at 34°C for up to 3 weeks. Samples were checked periodically for growth of *B. burgdorferi* by darkfield microscopy and C-chip disposable hemocytometers. One large square was observed for each sample which equates to a detection threshold of 10,000 cells/ml. Tubes with active *B. burgdorferi* growth were frozen at -80°C and were designated as primary isolates. The *B. burgdorferi* lineages present in primary isolates were genotyped at the *ospC* locus by the Luminex *ospC* genotyping (LOG) assay as previously described [38].

Obtaining *B. burgdorferi* clones by limiting dilution

B. burgdorferi primary isolates were grown in BSK-H complete media supplemented with 50 ug/ml rifampicin, 20 ug/ml phosphomycin, and 2.5 ug/ml amphotericin B [37] and subsequently diluted in fresh media with the same antibiotics to a final concentration of 0.5 or 1 cell/ml. Afterwards, 200 ul of the 0.5 or 1 cell/ml culture was added to wells of a 96-well plate resulting in a theoretical value of 0.1 or 0.2 spirochetes per individual well. The 96-well plates were

incubated at 34°C and 5% CO2 and checked periodically by darkfield microscopy for up to 4 weeks. The entire volume from individual wells with *B. burgdorferi* growth was transferred to fresh BSK-H complete media, with the same antibiotics as above, in 15 ml tubes and incubated at 34°C for up to 4 weeks. Putative *B. burgdorferi* clones were then frozen at -80°C in 5 replicate 1.5 ml tubes. The *B. burgdorferi* clones were genotyped at the *ospC* locus by the LOG assay [38] and Sanger sequencing.

Growth curves of *B. burgdorferi* clones used in the serum sensitivity assay

A growth curve was performed for clones-A, -I, -G, -H, and -M by diluting the frozen stock to a starting concentration of ~100,000 cells/ml in BSK-H complete media in triplicate 1.5 ml tubes, incubating at 34°C, and enumerating cell density by darkfield microscopy every 2-4 days. The clones were grown in a similar fashion for the serum sensitivity assay and cultures were chosen during the mid-logarithmic growth phase.
