# Supplementary material for: Differential Resistance of Borrelia burgdorferi Clones to Human Serum-Mediated Killing Does Not Correspond to Their Predicted Invasiveness
Source: Pathogens. 2023 Oct 13;12(10):1238. doi: 10.3390/pathogens12101238 (PMC10609869; doi:10.3390/pathogens12101238)
Supplement: Supplementary file 1 [file pathogens-12-01238-s001.zip › File_S2.pdf]

Morphologically heterogenous and motile *B. burgdorferi* cells were observed during the 4-hour serum sensitivity assay but inclusion of these forms in a separate analysis did not change the general results. Our definition of cell viability was retention of characteristic shape and motility [39,40]. Most of the motile *B. burgdorferi* cells observed during the 4-hour serum sensitivity experiment were characterized by the classic spiral shape. Some *B. burgdorferi* cells were motile but exhibited an atypical cell morphology. By convention these atypical forms were scored as not viable and not included in the data presented in Figure 1 and Table 1. Representative images of a spirochete with normal cell morphology and typically encountered atypical cells are included in Figures S2-S5 . Images were visualized and captured using a Leica DM2500 darkfield microscope at either 200X or 400X magnification. The images have been subsequently edited and magnified to highlight the different forms encountered.

A repeat analysis was performed that considered all motile cells regardless of their morphology as viable. Percent survival was calculated as the number of viable cells after 4 hours divided by the number of viable cells at the 0-hour time point multiplied by 100. These percent survival values were log-transformed and significant differences in survival of the clones were detected using a two-way ANOVA and Tukey's multiple comparisons test in GraphPad Prism version 9.0.0. *P*-values of less than 0.05 were considered significant. In this analysis the survivorship of the clones incubated in human serum was more uniform and fewer significant differences were detected (Figure S6, Table S1). Inclusion of the atypical, motile cells did not change the initial conclusions. The invasive clones did not have significantly higher survivorship compared to the noninvasive clones, suggesting that differential complement-mediated killing is not a determinant of *B. burgdorferi* lineage invasiveness. Complement-mediated killing is

similar among humans since the clones survived at comparable levels when incubated in the different human sera.
