# Supplementary figures and images for: Differential Resistance of Borrelia burgdorferi Clones to Human Serum-Mediated Killing Does Not Correspond to Their Predicted Invasiveness
Source: Pathogens. 2023 Oct 13;12(10):1238. doi: 10.3390/pathogens12101238 (PMC10609869; doi:10.3390/pathogens12101238)

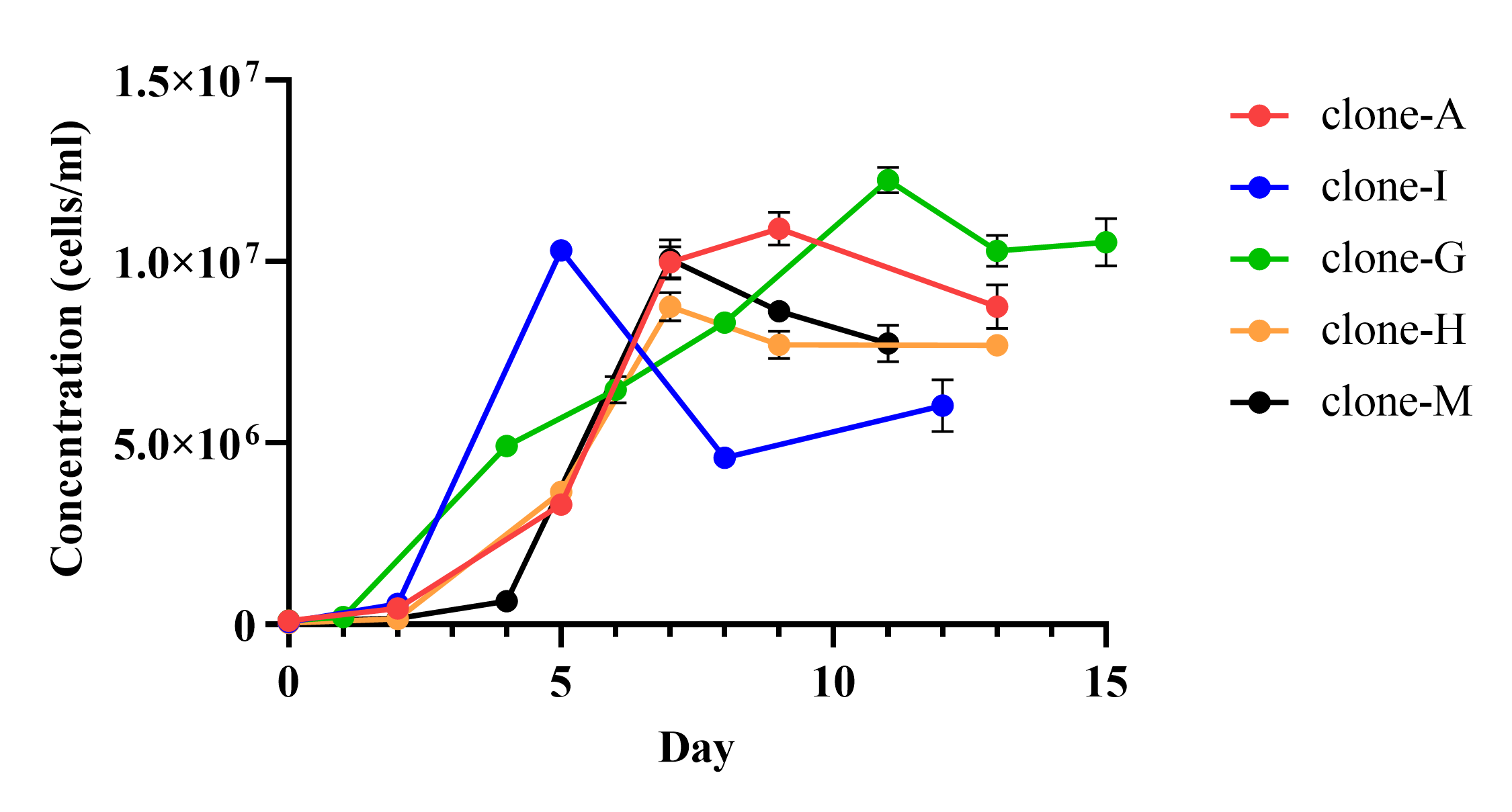

Supplement: Supplementary file 1 [file pathogens-12-01238-s001.zip › Figure_S1.tif]

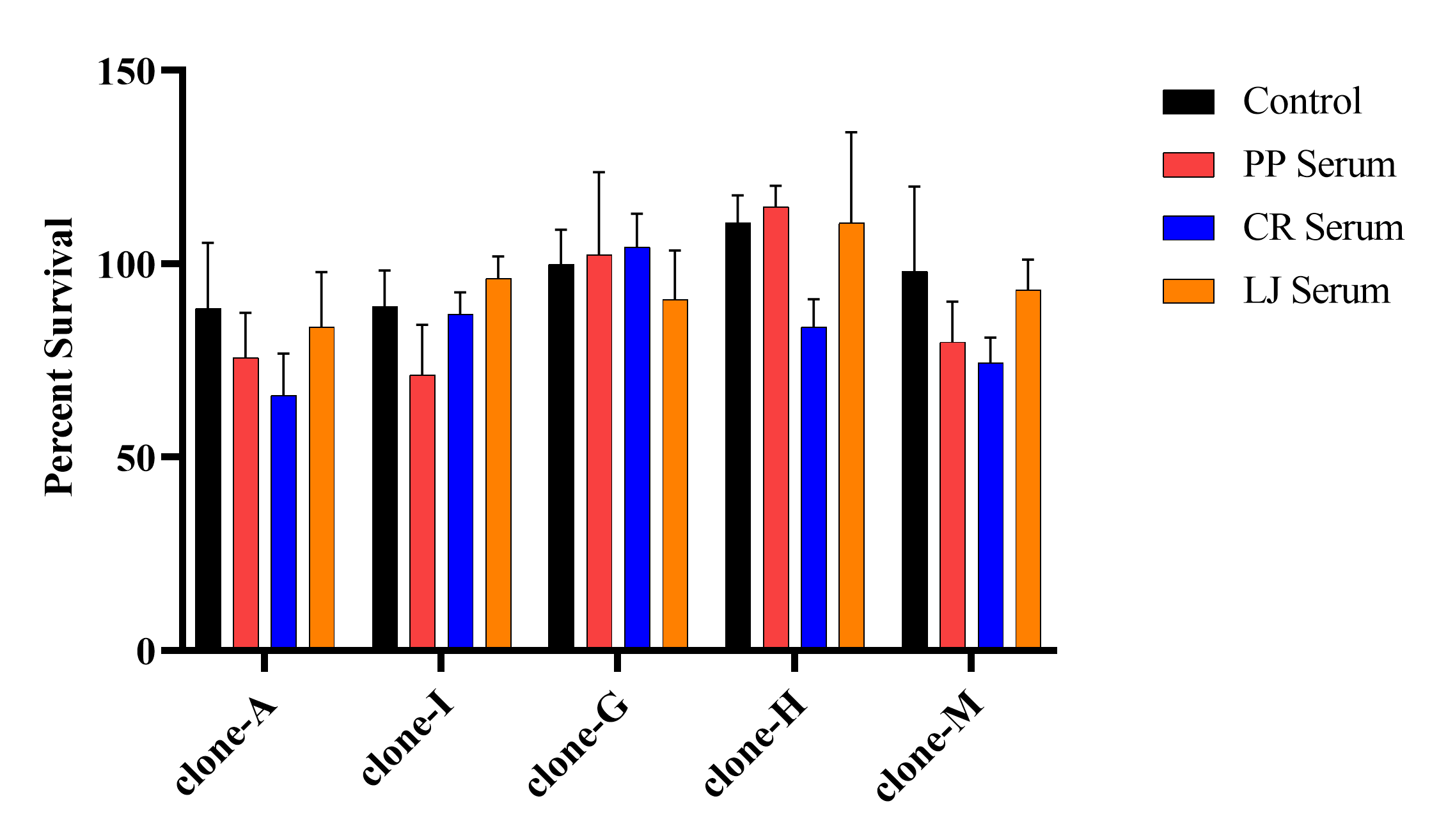

Supplement: Supplementary file 1 [file pathogens-12-01238-s001.zip › Figure_S6.tif]

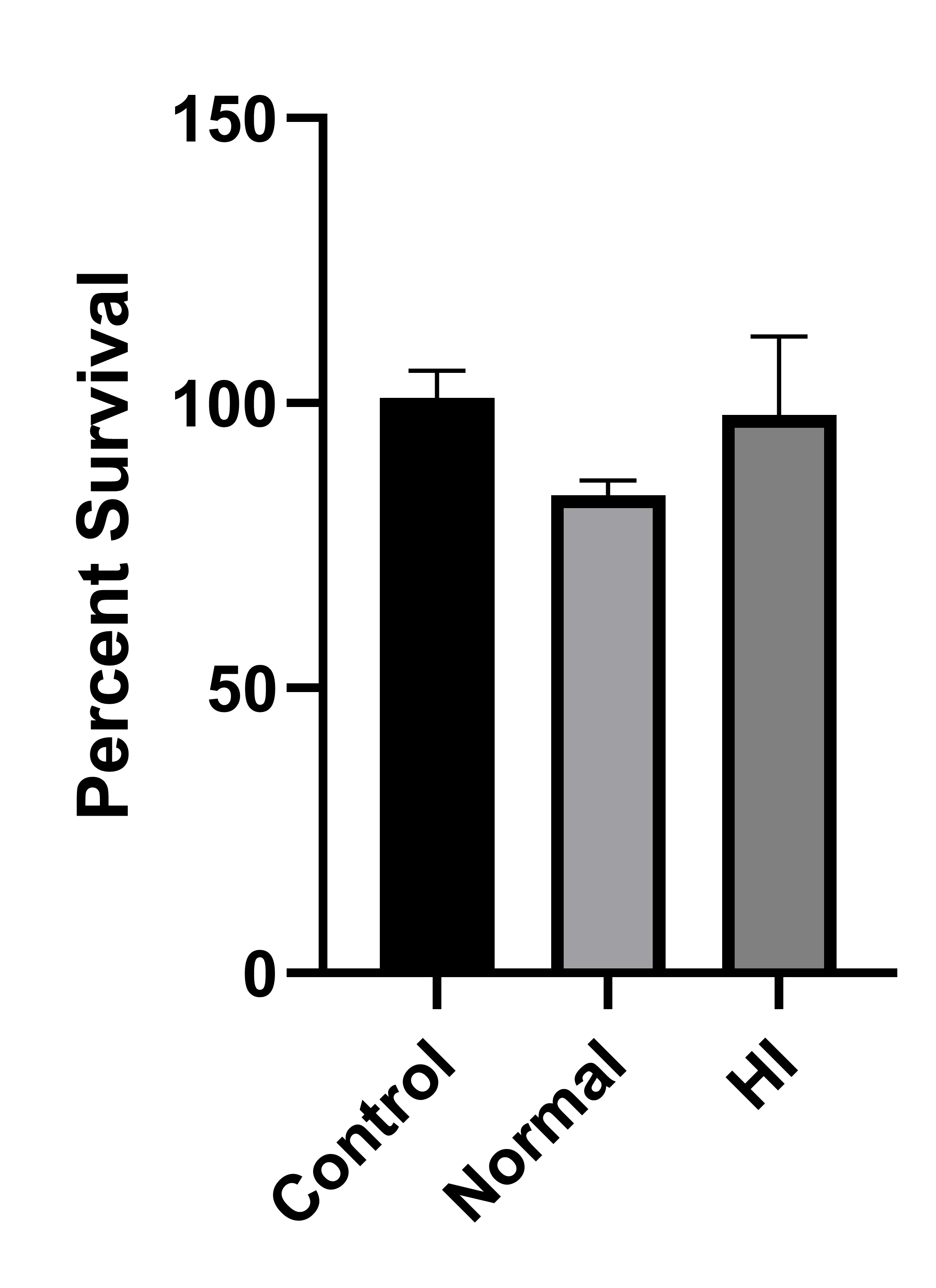

Supplement: Supplementary file 1 [file pathogens-12-01238-s001.zip › Figure_S7.tif]
